# Correlation of Pre-colonoscopy Blood Hemoglobin Levels With Significant Colorectal Pathology Among Fecal Immunochemical Test-Positive Patients

**DOI:** 10.7759/cureus.96710

**Published:** 2025-11-12

**Authors:** Abdulaziz Almasoud, Abdulrahman A Almalaq, Bayan Aldiebany, Ebtissam AlMeghaiseeb, Reem Alamro, Abdullah Albishi, Fuad Mohammad, Mohammed Al mutairi, Reem Alshowair, Mohamad Alharbi, Sayed Ammar, Abdullah Al mdani, Nasser Al Masri, Mutaz Abdelmahmoud, Malak Al Sudais, Jawaher Alanazi, Mohammed Almaghrabi, Abdulrahman Alrobayan

**Affiliations:** 1 Gastroenterology and Hepatology, Prince Sultan Military Medical City, Riyadh, SAU; 2 Gastroenterology, Prince Sultan Military Medical City, Riyadh, SAU; 3 Hepatology, Prince Sultan Military Medical City, Riyadh, SAU; 4 Gastroenterology, Prince Sultan Military Medical City, Riyadh , SAU

**Keywords:** advanced adenoma, anemia, colonoscopy, colorectal cancer screening, colorectal neoplasia, diagnostic yield, fecal immunochemical test (fit), hemoglobin, predictive factors, risk stratification

## Abstract

Background

Fecal immunochemical testing (FIT) is a widely implemented noninvasive screening tool for colorectal cancer (CRC). While FIT detects occult gastrointestinal (GI) bleeding, the relationship between systemic blood hemoglobin (Hb) levels and significant colorectal pathology (SCP) among FIT-positive patients remains uncertain. Identifying such an association could improve clinical triage and resource allocation in screening programs.

Methodology

This retrospective observational study was conducted at Prince Sultan Military Medical City, Riyadh, Saudi Arabia. All adults (≥18 years) who tested positive on FIT and subsequently underwent complete colonoscopy between December 2024 and September 2025 were included. Patients with incomplete colonoscopy, inadequate bowel preparation, missing Hb data, active non-colorectal GI bleeding, hematologic disorders, or prior colorectal surgery or malignancy were excluded. Pre-colonoscopy Hb was obtained within 12 weeks of colonoscopy. SCP was defined as tubular adenoma, tubulovillous adenoma, or adenocarcinoma. Hb levels were compared across pathology groups using the Kruskal-Wallis test, and anemia prevalence was analyzed using the chi-square test.

Results

Among 483 FIT-positive patients, 182 met the inclusion criteria. The mean age was 59.3 ± 11.8 years, and 55% were male. The median pre-colonoscopy Hb level was 13.9 g/dL (interquartile range (IQR) 12.7-15.0). Histopathology revealed tubular adenoma in 89 (48.9%), tubulovillous adenoma in 35 (19.2%), adenocarcinoma in 23 (12.6%), hyperplastic polyps in 16 (8.8%), and other benign lesions in 19 (10.4%). Median Hb differed slightly across groups, lowest in adenocarcinoma (12.8 g/dL) and highest in benign lesions (14.8 g/dL), but the difference was not statistically significant (*P* = 0.245). Overall, 139 (76%) patients, including 78% with adenocarcinoma, had normal Hb levels.

Conclusions

No significant correlation was observed between pre-colonoscopy blood hemoglobin and SCP among FIT-positive patients. Most individuals with advanced neoplasia had normal Hb concentrations, indicating that systemic hemoglobin is not a reliable predictor of malignancy in this population. Normal Hb should not be considered reassuring and must not delay colonoscopic evaluation following a positive FIT result.

## Introduction

Colorectal cancer (CRC) remains one of the most prevalent and lethal malignancies worldwide, ranking among the leading causes of cancer-related morbidity and mortality. According to the Global Burden of Disease Study 2019, the incidence and mortality of CRC have continued to rise in many parts of the world, particularly in countries undergoing rapid socioeconomic transition [1]. This growing burden underscores the importance of effective screening strategies that can detect premalignant lesions and early-stage cancers before clinical presentation.

Screening is central to CRC prevention and early diagnosis. Among the available screening modalities, fecal immunochemical testing (FIT) has emerged as the most practical and widely adopted non-invasive approach for population-level programs. FIT quantitatively measures human hemoglobin (Hb) in stool and has largely replaced guaiac-based fecal occult blood testing because of its higher sensitivity and specificity for lower gastrointestinal (GI) bleeding [2]. Multiple systematic reviews and guideline updates, including the U.S. Preventive Services Task Force evidence report, have demonstrated that FIT-based screening effectively reduces CRC incidence and mortality when applied at the population level [3].

Recent advances in screening strategies have focused on optimizing test performance and improving participation. Innovations in test design, risk-stratified screening intervals, and integration with other modalities such as colonoscopy have further enhanced diagnostic yield [4]. While colonoscopy remains the gold standard for detection and removal of adenomatous polyps, FIT provides a reliable, cost-effective alternative that maintains high acceptability and adherence rates [5].

Diagnostic accuracy of FIT varies depending on lesion location and disease stage, but studies consistently show excellent sensitivity for left-sided and early-stage cancers [6]. By detecting occult GI bleeding, FIT plays a pivotal role in identifying high-risk individuals who warrant timely colonoscopic evaluation.

However, only a limited number of studies have examined whether pre-colonoscopy systemic blood Hb levels are associated with significant colorectal pathology (SCP) among FIT-positive individuals. While prior research has primarily focused on fecal Hb concentration as a predictor of neoplasia [7-9], few investigations have evaluated the relationship between blood Hb and colonoscopic findings [10-12]. Identifying such an association could improve clinical risk stratification and guide prioritization for colonoscopy in resource-limited settings. Therefore, this study aimed to assess the association between pre-colonoscopy blood Hb levels and SCP among FIT-positive patients.

## Materials and methods

Study design and setting

This retrospective observational study was conducted at the Gastroenterology Department, Prince Sultan Military Medical City (PSMMC), Riyadh, Saudi Arabia. The study analyzed data from patients who tested positive on FIT and subsequently underwent colonoscopy between December 2024 and September 2025. The study was designed to evaluate the correlation between pre-colonoscopy blood Hb levels and the presence of SCP among FIT-positive individuals.

Study population

All adult patients aged 18 years and older with a positive FIT result who underwent complete colonoscopy with adequate bowel preparation were included. Patients were excluded if they had incomplete colonoscopy, inadequate bowel preparation (Boston Bowel Preparation Scale ≤6), missing pre-colonoscopy Hb data, non-diagnostic or unavailable histopathology, active GI bleeding unrelated to colorectal pathology (e.g., upper GI bleeding, hemorrhoidal bleeding, or angiodysplasia), known hematologic disorders, prior colorectal surgery or malignancy, or received blood transfusion or iron therapy between FIT testing and colonoscopy. Exclusions were verified through a detailed review of electronic medical records, endoscopy reports, and laboratory data to ensure accurate application of eligibility criteria and to minimize potential confounding factors affecting systemic Hb levels.

Data collection and variables

Data were retrieved from the electronic medical records and endoscopy reporting system at PSMMC. For each patient, the following variables were collected: demographic data (age and sex); laboratory data, including pre-colonoscopy blood Hb level (g/dL) obtained within 12 weeks before colonoscopy; and endoscopic and histopathologic findings, including the presence and type of colorectal lesions. Colonoscopy findings were categorized as follows: SCP, including tubular adenoma, tubulovillous adenoma, or adenocarcinoma; and non-SCP, including hyperplastic polyps, inflammatory lesions, or normal mucosa.

Definitions

FIT-positive: defined as a fecal Hb concentration above the institutional cutoff for positivity as determined by the PSMMC screening program.

Anemia: defined according to World Health Organization (WHO) criteria as Hb <13.0 g/dL in males and <12.0 g/dL in females.

Pre-colonoscopy blood Hb: the most recent Hb measurement obtained before the colonoscopic examination.

Outcomes

The primary outcome of interest was the presence of SCP on colonoscopy and histopathology. The main predictor variable was pre-colonoscopy blood Hb level (g/dL).

Secondary analyses included evaluating differences in anemia prevalence between SCP and non-SCP groups.

Statistical analysis

All analyses were performed using IBM SPSS Statistics for Windows, version 26.0 (IBM Corp., Armonk, NY). Continuous variables were expressed as mean ± standard deviation (SD) or median with interquartile range (IQR), depending on distribution. Categorical variables were summarized as frequencies and percentages.

Comparisons of Hb levels across multiple pathology groups were performed using the Kruskal-Wallis test, while categorical comparisons (e.g., anemia status) were conducted using the chi-square test. Statistical significance was defined as *P* < 0.05.

Ethical considerations

The study protocol was reviewed and approved by the Institutional Review Board of PSMMC. All patient data were anonymized before analysis. The requirement for informed consent was waived due to the retrospective nature of the study. The study was conducted in accordance with the ethical standards outlined in the Declaration of Helsinki.

## Results

Patient cohort description

During the study period, a total of 483 patients tested positive on FIT. After applying the predefined inclusion and exclusion criteria, 182 patients were eligible and included in the final analysis.

The mean age of the included patients was 59.3 ± 11.8 years, and 55% were male. The overall median pre-colonoscopy hemoglobin level was 13.9 g/dL (IQR 12.7-15.0). Histopathologic evaluation of colonoscopic findings revealed that tubular adenoma was the most common lesion, identified in 89 patients (48.9%), followed by tubulovillous adenoma in 35 patients (19.2%) and adenocarcinoma in 23 patients (12.6%). Hyperplastic polyps were detected in 16 patients (8.8%), while other benign lesions, including inflammatory polyps, mucosal prolapse, and non-neoplastic colonic mucosa, were observed in 19 patients (10.4%).

Across all pathology categories, there was substantial overlap in hemoglobin levels, and no statistically significant difference was observed among groups (*P* = 0.245, Table 1). Patients with adenocarcinoma demonstrated slightly lower median hemoglobin values compared to those with adenomatous or benign lesions; however, the majority remained within the normal range. As shown in Table 2, most patients with advanced colorectal pathology, including 78% of those with adenocarcinoma and 74% with tubulovillous adenoma, had normal pre-colonoscopy hemoglobin levels. These findings indicate that systemic blood hemoglobin concentration is not a reliable marker of SCP in FIT-positive patients and that normal hemoglobin should not delay or deprioritize colonoscopic evaluation following a positive FIT result.

**Table 1:** Comparison of pre-colonoscopy hemoglobin levels across pathology types. Data are presented as *n* (%), mean ± standard deviation (SD), and median (interquartile range (IQR)). The Kruskal-Wallis test was used to compare median hemoglobin levels among pathology types (*P* = 0.245). Median hemoglobin levels differed slightly among pathology categories, with the lowest values observed in adenocarcinoma and the highest in benign lesions. However, these differences did not reach statistical significance (*P* = 0.245). *The Kruskal-Wallis test comparing median hemoglobin levels among all pathology groups.

Pathology type	*n* (%)	Mean ± SD (g/dL)	Median (IQR) (g/dL)	*P*-value*
Adenocarcinoma	23 (12.6)	12.90 ± 2.01	12.8 (11.3-14.3)	
Tubular adenoma	89 (48.9)	14.18 ± 1.73	14.2 (13.0-15.3)	
Tubulovillous adenoma	35 (19.2)	14.09 ± 1.59	14.0 (12.8-14.9)	
Hyperplastic polyp	16 (8.8)	13.85 ± 1.38	14.2 (13.0-14.9)	
Other (inflammatory / non-neoplastic)	19 (10.4)	14.80 ± 1.96	14.8 (13.4-15.9)	
Total	182 (100)	-	-	0.245

**Table 2:** Distribution of normal vs. low hemoglobin by pathology types. Data are presented as number (percentage). Low hemoglobin (Low Hb) was defined as <13 g/dL in males or <12 g/dL in females, according to WHO criteria. Data are presented as *n* (%), mean ± standard deviation (SD), and median (interquartile range (IQR)). The chi-square test was used to compare distributions across pathology groups (*P* = 0.245). Across all pathology categories, approximately three-quarters (76%) of patients had normal hemoglobin levels, including nearly four out of five individuals with adenocarcinoma. Although the mean hemoglobin level was slightly lower in those with malignant lesions, most patients with significant colorectal pathology were not anemic. These findings confirm that blood hemoglobin level is not a reliable indicator of advanced colorectal pathology in FIT-positive individuals. FIT, fecal immunochemical testing

Pathology type	*n* (%)	Normal Hb (%)	Low Hb (%)	Comments
Adenocarcinoma	23 (12.6)	18 (78)	5 (22)	Lowest median Hb (12.8 g/dL)
Tubular adenoma	89 (48.9)	65 (73)	24 (27)	Most common lesion
Tubulovillous adenoma	35 (19.2)	26 (74)	9 (26)	Moderate variation
Hyperplastic polyp	16 (8.8)	13 (81)	3 (19)	Mostly normal Hb
Other (inflammatory/non-neoplastic)	19 (10.4)	17 (89)	2 (11)	Combined benign group
Total	182 (100)	139 (76)	43 (24)	-

## Discussion

This study evaluated the relationship between pre-colonoscopy blood Hb levels and SCP among patients who tested positive on FIT. The principal finding was the absence of a significant correlation between systemic Hb concentration and the presence of advanced colorectal lesions. Although patients with adenocarcinoma had slightly lower median Hb values, most remained within the normal range. These results indicate that anemia is not a consistent feature among FIT-positive individuals with SCP, suggesting that blood Hb lacks discriminatory value in this setting.

Previous research has consistently highlighted the predictive value of fecal Hb rather than systemic Hb. A recent meta-analysis demonstrated a clear dose-response relationship between fecal Hb concentration and colorectal neoplasia risk [7]. Other studies confirmed that increasing fecal Hb levels were associated with advanced neoplasia and CRC detection during colonoscopy [8-10]. In contrast, systemic blood Hb appears to be influenced by multiple physiological factors and does not accurately reflect localized bleeding from colorectal lesions [11]. Similar observations were reported in prior studies, which demonstrated that low Hb was not a reliable predictor of malignancy among FIT-positive populations [12].

Several investigators have explored whether combining FIT with hematologic indices or iron deficiency anemia improves diagnostic accuracy. Withrow et al. reported that adding routine blood tests to FIT marginally enhanced colorectal cancer risk stratification [13]. Similarly, Pham et al. demonstrated that combining FIT results with iron deficiency anemia improved the detection of advanced neoplasia in asymptomatic patients [14]. However, systematic reviews concluded that while these models may improve triage in selected settings, their performance remains variable and context-dependent [15,16]. A recent retrospective study also showed that integrating fecal Hb, anemia status, and age can refine CRC risk prediction, though normal Hb levels were still observed in a substantial proportion of cancer cases [16]. Taken together, these data align with our findings that normal systemic Hb should not be considered reassuring following a positive FIT result.

From a pathophysiologic standpoint, the presence of normal systemic Hb in patients with advanced pathology can be explained by the often intermittent and microscopic nature of bleeding in early-stage or right-sided colorectal lesions. Compensatory erythropoiesis, chronicity of blood loss, and varying iron absorption may maintain normal Hb levels despite ongoing occult bleeding. Therefore, the diagnostic value of systemic anemia is limited, and reliance on its presence may delay timely colonoscopy, counteracting the intent of population-based FIT programs [13,15,16].

These findings have important implications in both global and regional contexts. CRC remains the third most common malignancy worldwide and the second leading cause of cancer-related mortality [17]. In Saudi Arabia, recent data indicate a steady rise in CRC incidence and a trend toward younger age at diagnosis [18,19]. Furthermore, global analyses have identified early-onset CRC as an emerging health challenge, with increasing incidence across multiple regions [20]. Given these epidemiologic patterns, optimizing screening efficiency is critical. FIT-based programs play a pivotal role in early detection, and the current study reinforces that all FIT-positive individuals warrant prompt colonoscopic evaluation regardless of their Hb status.

The strengths of this study include a well-defined cohort of FIT-positive patients, histopathologic confirmation of all lesions, and a comprehensive review of pre-colonoscopy laboratory data. Limitations include the retrospective single-center design, potential referral bias, and absence of detailed iron indices or ferritin levels in all cases, which could have provided a more granular assessment of iron deficiency anemia.

In conclusion, our findings demonstrate that normal pre-colonoscopy Hb levels are common among FIT-positive patients with SCP, including adenocarcinoma. Blood Hb concentration is therefore not a reliable predictor of malignancy in this context. FIT positivity alone should remain the primary determinant for diagnostic colonoscopy referral, and normal systemic Hb should not delay or deprioritize evaluation. Future prospective studies integrating fecal Hb quantification with hematologic and demographic variables may further refine individualized risk assessment in CRC screening.

## Conclusions

In this study of FIT-positive patients undergoing colonoscopy, no significant correlation was observed between pre-colonoscopy blood Hb levels and the presence of SCP. Most patients with advanced neoplasia, including adenocarcinoma, had normal Hb concentrations. These findings demonstrate that systemic Hb is not a reliable predictor of malignancy in FIT-positive individuals. Normal Hb should not be considered reassuring and must not delay colonoscopic evaluation. Further prospective studies are warranted to explore integrated models combining fecal Hb, demographic, and laboratory parameters to enhance risk stratification in CRC screening programs.
